# Genome Dynamics of *Escherichia coli* during Antibiotic Treatment: Transfer, Loss, and Persistence of Genetic Elements *In situ* of the Infant Gut

**DOI:** 10.3389/fcimb.2017.00126

**Published:** 2017-04-12

**Authors:** Andreas Porse, Heidi Gumpert, Jessica Z. Kubicek-Sutherland, Nahid Karami, Ingegerd Adlerberth, Agnes E. Wold, Dan I. Andersson, Morten O. A. Sommer

**Affiliations:** ^1^Novo Nordisk Foundation Center for Biosustainability, Technical University of DenmarkLyngby, Denmark; ^2^Department of Clinical Microbiology, Hvidovre University HospitalHvidovre, Denmark; ^3^Department of Medical Biochemistry and Microbiology, Uppsala University Biomedical CentreUppsala, Sweden; ^4^Department of infectious Diseases, University of Gothenburg, Sahlgrenska AcademyGothenburg, Sweden

**Keywords:** *Escherichia coli*, genome evolution, virulence plasmid dynamics, plasmid persistence, horizontal gene transfer, antibiotic treatment, urinary tract infections, infant gut

## Abstract

Elucidating the adaptive strategies and plasticity of bacterial genomes *in situ* is crucial for understanding the epidemiology and evolution of pathogens threatening human health. While much is known about the evolution of *Escherichia coli* in controlled laboratory environments, less effort has been made to elucidate the genome dynamics of *E. coli* in its native settings. Here, we follow the genome dynamics of co-existing *E. coli* lineages *in situ* of the infant gut during the first year of life. One *E. coli* lineage causes a urinary tract infection (UTI) and experiences several alterations of its genomic content during subsequent antibiotic treatment. Interestingly, all isolates of this uropathogenic *E. coli* strain carried a highly stable plasmid implicated in virulence of diverse pathogenic strains from all over the world. While virulence elements are certainly beneficial during infection scenarios, their role in gut colonization and pathogen persistence is poorly understood. We performed *in vivo* competitive fitness experiments to assess the role of this highly disseminated virulence plasmid in gut colonization, but found no evidence for a direct benefit of plasmid carriage. Through plasmid stability assays, we demonstrate that this plasmid is maintained in a parasitic manner, by strong first-line inheritance mechanisms, acting on the single-cell level, rather than providing a direct survival advantage in the gut. Investigating the ecology of endemic accessory genetic elements, in their pathogenic hosts and native environment, is of vital importance if we want to understand the evolution and persistence of highly virulent and drug resistant bacterial isolates.

## Introduction

The human gut is home to a dense microbial ecosystem, the human gut microbiota, playing an important role in human health and physiology (Marchesi et al., 2015). As a commensal constituent of the gut microbiota in warm-blooded animals, *Escherichia coli* is highly adapted to the gut and colonizes the gastrointestinal tract within the first hours of life (Drasar and Hill, 1974). However, some environmental and commensal *E. coli* isolates have acquired genetic factors that allow them to cause disease within the digestive tract or when transferred to other body sites such as the blood, brain, and urinary tract (Smith et al., 2007). While diarrheagenic *E. coli* are a common cause of gastro intestinal infections in third world countries and travelers, extraintestinal pathogenic *E. coli* (ExPEC) are facultative pathogens that reside in the human gut microbiota but occasionally establish in extra-intestinal body sites (Köhler and Dobrindt, 2011). Here, urinary tract infections casued by ExPEC are among the most common bacterial infections in developed countries, were patients are often infected via transmission of strains from their own intestinal flora to their urinary tract (Foxman, 2010).

The broad adaptation of *E. coli* to the gut environment and extraintestinal body sites is reflected in the remarkable genetic diversity within the species. This genetic flexibility is largely facilitated by horizontal gene transfer (HGT) of accessory genetic elements including plasmids and phages (Brzuszkiewicz et al., 2009). These elements are widely present within the gut microbiota and can provide their bacterial hosts with antibiotic resistance or virulence factors (Salyers et al., 2004; Sommer et al., 2010). Acquiring virulence genes might not only influence the risk and severity of infections caused by the pathogen, but has also been suggested to assist in the general persistence of commensal bacterial strains of the gut (Diard et al., 2010; Chen et al., 2013). Indeed, virulence determinants such as those involved in adhesion, biofilm formation and iron acquisition correlate with prolonged colonization in the digestive tract (Adlerberth et al., 1998; Nowrouzian et al., 2003).

Recent studies into the dynamics of clinical bacterial genomes at genomic resolution have been carried out with time-series sampling and underlines the high plasticity of plasmids and their host associations *in situ* (Conlan et al., 2014, 2016). Conjugative plasmids are of particular interest, as they are the main vehicles of HGT in *E. coli*, playing an essential role in the adaptation toward antibiotics or specific host niches (Johnson and Nolan, 2009; Norman et al., 2009).

Whereas, much effort has been devoted to study the survival conditions of plasmids *in vitro* (Slater et al., 2008) our knowledge on the behavior of plasmids *in situ* of their native hosts and natural environment is limited (Karami et al., 2007; Conlan et al., 2014, 2016). In order for a plasmid to persist in the long term, it needs to either be stably segregated upon cell division, confer a fitness advantage to its host, or transfer at high enough rates to compensate the lack of the latter two (Simonsen, 1991; Slater et al., 2008). As most plasmids do not exhibit sufficient rates of transfer to survive without selection, stable inheritance, and adaptive traits are key to their long term survival (Simonsen, 1991).

To elucidate the genome dynamics of *E. coli* in its native environment of the gut, we genome sequenced individual *E. coli* isolates over the first year of an infant's life. We conduct *in vitro* and *in vivo* competition assays to elucidate the selective drivers of the observed dynamics, and gain a deeper understanding of the endemic mobile elements contributing to the dissemination of virulence and antibiotic resistance factors.

## Materials and methods

### Genome sequencing of *E. coli* lineages

The strains were isolated and typed as part of a previous study by Karami et al. (2007). These were cultured in LB broth and genomic DNA was isolated using an UltraClean Microbial DNA Isolation Kit (MoBio Laboratories, Inc., California). Sequencing libraries were prepared using the TruSeq and Nextera XT (Illumina, California) protocols. Illumina HiSeq sequencing was performed by Partners HealthCare Center for Personalized Genetic Medicine (Cambridge, Massachusetts).

### Sequence analysis

Genomes for each sequenced isolate were assembled using Velvet (v1.2.10; Zerbino and Birney, 2008) and annotated via RAST (Aziz et al., 2008). Reads from the isolates were mapped onto the reference, e.g., earliest isolated, genome via Bowtie2 (2.1.0; Langmead et al., 2009), and single nucleotide polymorphisms (SNPs) were enumerated via SAMTools (0.1.19; Li et al., 2009). The SNP threshold was set to include SNPs with a phred score of above 30 and at least 90% of the high-quality reads at the site as the variant. Additionally, to ensure that all isolates within a lineage consisted of the same genomic content as the representative isolates, genomic areas lacking mapped read coverage were identified using BEDTools (2.18.2; Quinlan and Hall, 2010).

The pNK29 plasmid was assembled into a circular plasmid with aid from plasmid alignments produced using MUMer (Kurtz et al., 2004). Contigs belonging to the pNK29 antibiotic resistance plasmid were first identified in lineage B as the new genetic material of the isolate at 32 days, and then used to identify the corresponding contigs in the lineage A genome. The RAST annotations for this plasmid were refined based on homologous genes in pOLA52 (NC_010378.1) that were either missing or incorrect in pNK29.

### Plasmid identification and comparison

Other plasmids were identified by first separating contigs based coverage to infer copy-number relative to genomic contigs and then by grouping contigs together with similar abundances. The average coverage of each contig was determined using BEDTools (Quinlan and Hall, 2010). Plasmid incompatibility grouping was done using the PlasmidFinder tool (Carattoli et al., 2014). Homologous previously sequenced plasmids were identified using BLAST and the NCBI nt database (Altschul et al., 1990). Circular plasmid diagrams were created using the BLAST ring image generator (BRIG; Alikhan et al., 2011).

For pNK29-2, blastn searches of the plasmid contigs revealed 14 plasmids with very high identity (99%) and hits with a pNK29-2 coverage of >97% where selected (**Figure 3** and Table S4). As an exception, pECO-bc6 was also included despite its lower coverage (88%) to illustrate deletion of plasmid accessory genes flanked by inverted repeats. The EasyFigure software was used for linear comparison of plasmid sequences displayed in **Figure 4** (Sullivan et al., 2011).

The core genome of the *E. coli* hosts listed in **Figure 3** was estimated using ROARY via annotations from PROKKA (Seemann, 2014; Page et al., 2015). The aligned, ungapped core genome was used to construct a maximum likelihood phylogenetic tree using the RAxML software (Stamatakis, 2014). MLST types were assigned using MLSTfinder (Larsen et al., 2012), and *fimH* types were assigned using the sequences referred to by Dias et al. (2010).

### Strain tagging and pNK29-2 plasmid curing

Lineage A and B strains isolated at the first time point were tagged with antibiotic resistance markers to allow quantification during competitive fitness experiments, plasmid loss experiments, assessment of conjugation ability, and plasmid curing. Resistance cassettes conferring resistance to Chloramphenicol and Kanamycin respectively were amplified from cloning vectors of the pZ system (Lutz and Bujard, 1997) and inserted into the chromosomal *araB* gene of the Lineage A and B strains using the Lambda Red recombineering system of pTKRED (Kuhlman and Cox, 2010). The following regions of homology were used for insertions into *araB*: 5′-GTAGCGAGGTTAAGATCGGTAATCACCCCTTTCAGGCGTTGGTTAGCGTT-3′ and 5′-GCCTAACGCACTGGTAAAAGTTATCGGTACTTCCACCTGCGACATTCTGA-3′.

The pNK29-2 plasmid was tagged with the Sh *ble* Zeocin resistance gene in a transposase gene located at 61 kb using the following homology ends: 5′-CTTCGGGAACGCTGTAACGATTACCACCAACCTCGATATAGCTGTCCCGG-3′ and 5′-TAACAACGGGAAAGTCGTGTTCAACTCCGGATTCCTGTTGCTGGCCGACC-3′.

To cure pNK29-2 we disrupted the *stbA* gene of the *stbAB* stability operon with a Kanamycin resistance marker using the following homology ends: 5′-CATAAATGTGATGTGTGAAGTATGATGATATTTTGACACGGTAACCTGAG*TAGGGATAACAGGGTAAT-*3′ and 5′-TTCATTTTAAGACGCACATCATTCATTGCCTCCTGCACCGAATCAGTAGC *TAGGGATAACAGGGTAAT-*3′. These contain the 18 bp I-SceI endonuclease site enabling *in situ* double digestion of the recombinant plasmid to enhance plasmid loss via induction of the I-SceI endonuclease from pTKRED. Following recombineering, recombinants were selected on Kanamycin containing plates incubated at 30°C to retain the pTKRED plasmid. These were verified with PCR to confirm insertion into the *stbA* gene. Verified colonies were inoculated directly into LB medium containing 0.5% w/v L-arabinose and grown at 30°C for 2 h to induce expression of the pTKRED encoded I-SceI endonuclease and subsequently switched to 42°C for 3 h to allow curing of the temperature sensitive pTKRED vector. The culture was diluted and plated on LB to obtain single colonies that were confirmed for plasmid curing by the absence of growth on Kanamycin (pNK29-2) and Spectinomycin (pTKRED). The curing of pNK29-2 was validated by PCR using primers targeting two distinct loci of the pNK29-2 backbone: Upstream *stb*: 5′-CTCAACAAGGGTTATTGC-3′, downstream *stb*: 5′-GAATGGCAAATGAAACG-3′ and Upstream 61 kb transposase: 5′-GAATGGCAAATGAAACG-3′, downstream 61 kb transposase: 5′-AGAAGGCTGCGGTGCTGAAG-3′. The plasmid-cured variant of the lineage A strain was re-transformed with the Zeocin tagged pNK29-2 plasmid to control for potential effects of the curing process.

### Conjugative transfer assessment

In order to test the ability of pNK29-2 to conjugate, outgrown over-night (O/N) cultures as well as exponentially growing cultures of the lineage A strain and *E. coli* MG1655::tetA was mixed equally and incubated O/N. Incubations were done at 37°C and 30°C on a solid agar surface as well as in liquid cultures without shaking. Additional tagging at the 32 and 26 kb positions were carried out to ensure that the initial insertion at the 61 kb position of pNK29-2 was not the cause of the dysfunctional conjugation ability.

### *In vitro* competition, growth rate, and plasmid loss assays

Two O/N cultures were diluted to the same OD and mixed 1:1 in LB medium. The competition was carried out in 1.5 ml cultures and a volume of 1.5 μl was transferred to a fresh well every 20 h. From OD measurements the number of generations was estimated to ~10 generations/transfer. The ratio of the competitors was determined as the fraction of colonies on Chloramphenicol agar plates compared to plates containing Kanamycin. Plasmid loss was assessed by comparing plate-counts of at least 100 colonies on LB and Zeocin agar plates. In addition, colonies from LB plates were streaked on Zeocin containing plates and PCR (using the primers listed in the plasmid curing section) was performed on a subset of colonies to verify plasmid presence. Competitions in iron-limited medium was carried out as for the LB competitions described above, except that M9 medium [M9 minimal medium (standard), 2010] with 5 μM FeCl_3_ was used instead or LB. OD measurements of growth rates in M9 5 μM FeCl_3_ were conducted in 96-well plates containing 150 μl medium/well using a *ELx808* plate reader (BioTek, USA). Breathe-Easy (Sigma-Aldrich) film was applied to minimize evaporation during measurements. OD at 600 nms was measured with 5-min intervals for 24 h and incubated with shaking at 37°C between measurements.

### *In vivo* competition experiments

Female BALB/c mice (5–6 weeks old) were used in all *in vivo* studies (Charles River Laboratories, distributed by Scanbur). All mice were pre-treated orally with streptomycin as described previously (Lasaro et al., 2014). Briefly, streptomycin sulfate salt (Sigma-Aldrich) was added to the drinking water at 5 g/L, along with 5 g/L of glucose, to enhance taste, for 72 h followed by 24 h of fresh water (no drug or glucose) to allow the streptomycin to clear the animal's system prior to infection. A single colony of each *E. coli* strain was grown in LB shaking overnight at 37°C. Cells were pelleted, washed once in PBS and re-suspended in PBS (13 mM phosphate with 137 mM NaCl at pH 7.4), and then mixed in a 1:1 ratio of *E. coli* lineage A isolate with pNK29-2 to the cured variant. Fifteen mice that were pre-treated with streptomycin followed by 24 h without drug were administered 100 μL containing 2 × 10^8^ CFU of this 1:1 *E. coli* mixture by oral gavage. Feces was collected at days 2, 4, 7, and 12 post-infection. Additionally, on day 12 following termination of the experiment, a segment of the small intestine was removed. The feces and small intestine segment were homogenized in PBS, serially diluted, and equal amounts were plated on LA-Cam (25 μg/ml chloramphenicol, selecting for the pNK29-2 containing strain) and LA-Kan (50 μg/ml kanamycin, selecting for the cured strain). Following overnight incubation at 37°C, CFUs were enumerated and subsequently replica plated from both LA-Cam and LA-Kan to LA-Zeo (40 ug/ml zeocin) to screen for the presence and transfer of the pNK29-2 plasmid containing a Zeocin-resistance marker. CFU values were normalized per gram of tissue (CFU/g). The competitive index was calculated by dividing the OUTPUT on days 2, 4, 7, and 12 (CamR CFU/g divided by KanR CFU/g) by the INPUT on day 0 (CamR CFU/g divided by KanR CFU/g). The input value was 1.04 indicating a 1:1.04 initial ratio of *E. coli* lineage A isolate with pNK29-2 relative to the cured *E. coli* lineage A isolate. The non-parametric Mann-Whitney *U*-Test was used to compare the sample populations. The *P-*values indicate the probability of falsely rejecting the null-hypothesis of equal population means.

### Ethics statement

Animal experiments were performed in accordance with national (regulation SJVFS 2012:26) and institutional guidelines. The Uppsala Animal Experiments Ethics Review Board in Uppsala, Sweden approved all mouse protocols undertaken in this study under reference no. 154/14. Animal experiments were performed at the Swedish National Veterinary Institute (SVA) in Uppsala, Sweden.

## Results

The current study material was obtained from an infant enrolled in the ALLERGYFLORA study with the original purpose of correlating the composition of the gut microbiota to the development of allergies later in life (Adlerberth et al., 2006).

This infant was subjected to long-term antibiotic treatment as a consequence of a urinary tract infection, and was selected for this study due to an observed change in the resistance profile of *E. coli* strains isolated from this infant (Karami et al., 2007).

**Figure 1:**
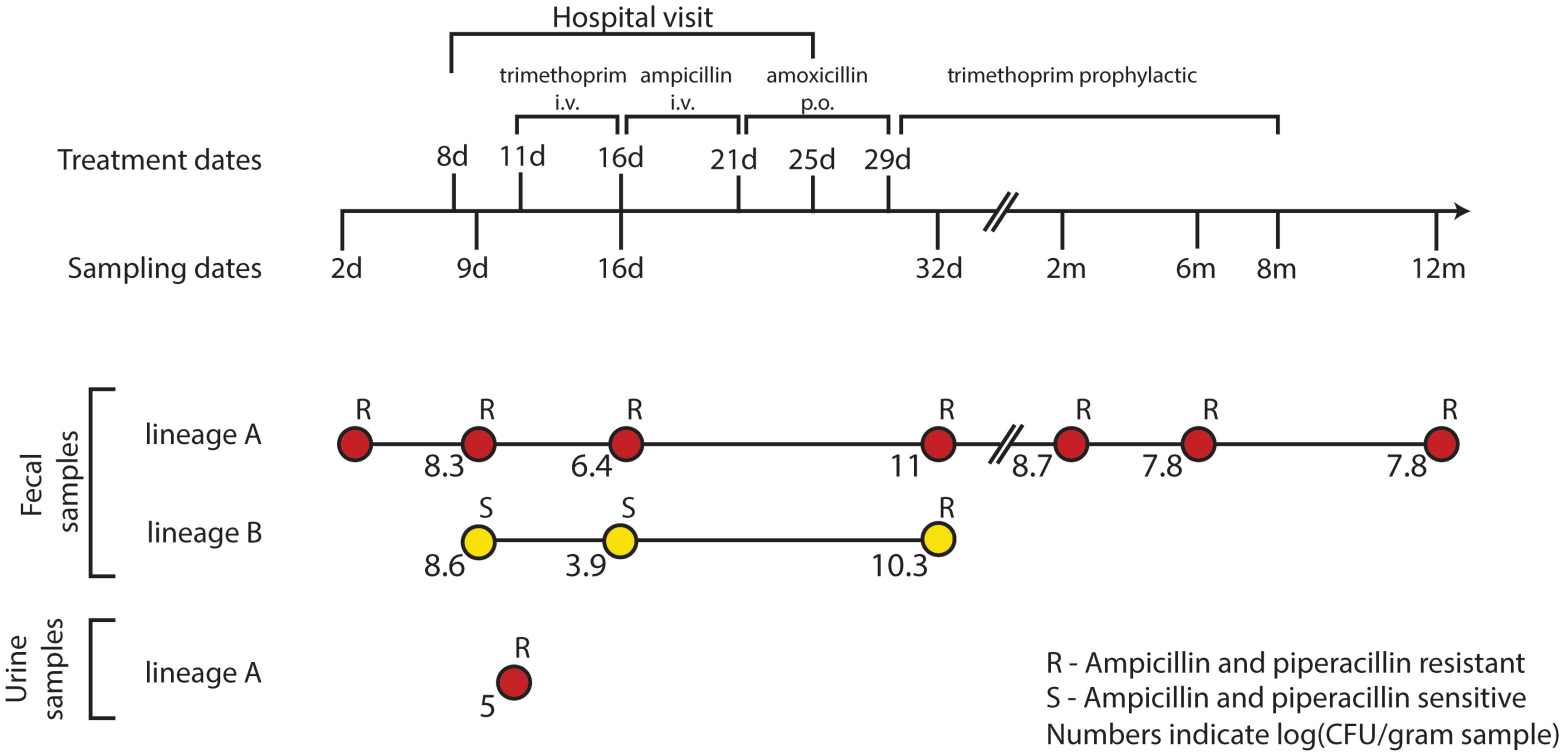
**Resistance profiles of co-existing ***E. coli*** lineages during the course of a urinary tract infection and antibiotic treatment**. *E. coli* isolates were obtained during the first 12 months of an infant's life. At 8 days of age, the infant was admitted to the hospital due to a urinary tract infection from which the lineage A clone was isolated at day 11. After 5 days of i.v. trimethoprim the treatment was switched to 5 days of i.v. ampicillin followed by an additional 8 days of oral (p.o.) amoxicillin. Trimethoprim was administered to prevent reoccurring infections for the following 7 months. Along the course of treatment, lineage B acquired a TEM-1b encoding IncX plasmid from lineage A; rendering both lineages resistant to the β-lactam treatment at day 32.

Fecal samples were obtained from the infant at 2, 9, 16, and 32 days, and 2, 6, and 12 months after birth (Figure 1). *E. coli* lineages were identified by morphological and biochemical characteristics as well as subsequent confirmation by PFGE and random amplified polymorphic DNA (RAPD) typing. The two main lineages were designated “A” and “B.” *E. coli* lineage A was recovered at all the sampling time points and lineage B was only transiently present in the samples collected from day 9 to 32 days of age (Karami et al., 2007; Figure 1). At 11 days of age, a UTI infection was diagnosed and trimethoprim administered intravenously (i.v.) for 5 days. However, due to the subsequent presence of enterococci in addition to *E. coli*, the antibiotic treatment was changed to i.v. ampicillin for 5 days followed by oral amoxicillin treatment for an additional 8 days. Lastly, the infant was administered trimethoprim prophylactically for the following 7 months (Figure 1).

### Transfer of an antibiotic resistance plasmid between two distinct *E. coli* lineages co-colonizing the infant gut

We sequenced the genomes of the *E. coli* isolates, obtained from the fecal and urine samples of the infant, which confirmed the lineages previously identified via RAPD and PFGE typing. Comparing the genomic similarity of the two lineages revealed that the lineages did indeed originate from two different strains; with the initial isolate of lineage A (4.91 Mb) sharing only 77% of lineage B's (5.45 Mb) genomic content.

**Figure 2:**
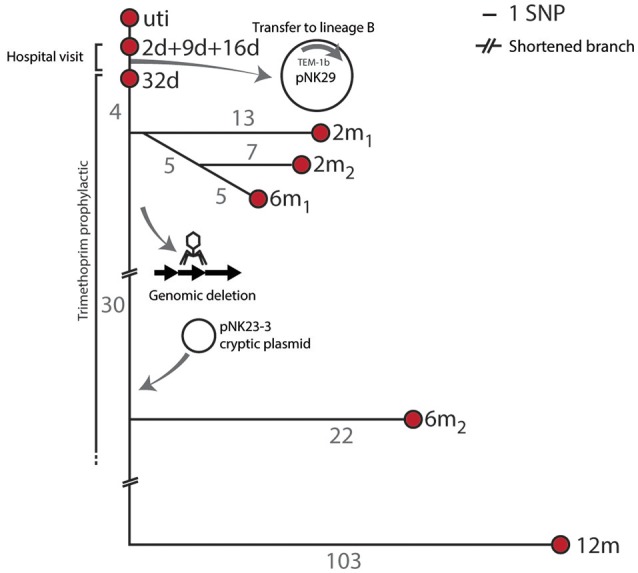
**SNP tree and genomic events of lineage A**. Branch-numbers show the amount of SNPs separating the isolates obtained from 2 days to 12 months after birth. The majority of samples were isolated during hospitalization and prophylactic treatment. No SNPs were detected between the isolates collected at days 2, 9, and 16, and only one SNP (in the blaTEM1b promoter) was detected between these isolates and the isolate collected at 32 days. Two isolates were included at the 2 months sampling point (2 m1 and 2 m2) and these shared 5 SNPs in common compared to the 32 days isolate. Two 6 month isolates were also included. The infant was subjected to antibiotic treatment from day 11 to 8 months after birth. Apart from SNP differences one sub-lineage had undergone major genomic changes by means of chromosomal deletions and acquisition of new plasmid DNA.

To assess the genomic divergence of the individual lineage A isolates, during the sampling period, a SNP-based phylogenetic tree was constructed (Figure 2, Table S1). Only one SNP was found when comparing the genomes of lineage A isolates collected at 2, 9, and 16 days to the UTI isolate (Figure 2). Given the very high sequence similarities between these isolates, lineage A colonizing the gut microbiota was assumed to be the cause of the UTI.

From the annotated genomes of lineage A and lineage B isolates, we identified several factors that could contribute to the pathogenicity of these strains. The genome of the uropathogenic lineage A encoded the type 1 fimbriae FimH among other adhesion factors (*AidA-I* and *yqi* encoded adhesions), siderophore (enterobactin and yersiniabactin) transporters and hemin receptors (TonB-system) as well as enterotoxins (*senB* and *vat*) and Hemolysin E. Although the lineage B isolates did not cause infection, its genomic content reveals similar virulence factors such as the type 1 fimbriae (*fimH*), serum survival factors (*iss*) and iron acquisition (aerobactin synthesis and transport), but no enterotoxins.

While no SNPs were detected in the three isolates from lineage B, we report the sequence of pNK29, a 42.2 kb TEM-1b encoding conjugative IncX plasmid, that transferred from lineage A to lineage B *in situ* of the gut (Karami et al., 2007; Figure S1, Table S2). This plasmid was first detected in lineage B at 32 days, and coincided with high resistance toward ampicillin compared to earlier isolates (Figure 1). Similar conjugative plasmids of the IncX family are prevalent in pathogenic *E. coli*, as well as other Enterobacteriaceae isolated from humans and animals, playing an important role in the dissemination of antibiotic resistance genes (Norman et al., 2008; Toro et al., 2014).

Karami et al. reported an increase in lineage A counts from 10^6.4^ CFU/g fecal matter to a density of 10^11^ CFU/g as the infant was switched from trimethoprim to ampicillin and amoxicillin treatment during the UTI infection from day 16 to 32 (Karami et al., 2007). Such events can increase population size, and thus the probability of plasmid transfer and enrichment of pNK29 bearing cells. A similar increase in population counts from 10^3.9^ to 10^10.3^ CFU/g was observed for lineage B as a result of pNK29 acquisition and antibiotic selection (Figure 1).

Interestingly, pNK29-bearing lineage B isolates were no longer detected in the subsequent samples collected after cessation of amoxicillin treatment (Figure 1). Plasmids often impose a fitness cost upon first encounter with new host backgrounds which could render lineage B less fit in the absence of selection (Porse et al., 2016). Measuring the *in vitro* competitive fitness of the initial plasmid-carrying lineage B isolate revealed a burden of carriage (−4.9%, *sd* ± 4.1%, *P* = 0.046); indicating that a counterselection of linage B, due to plasmid invasion, might have taken place after discontinuation of amoxicillin treatment.

### Major genomic events of lineage a during gut colonization

Apart from the pNK29 plasmid, all isolates of lineage A carried a large virulence plasmid, designated pNK29-2, which was detected throughout the year of sampling. In addition to these two large plasmids, a novel plasmid-element was detected in the 6 m_2_ and 12 m isolates (Figure 2). This small (2,545 bp) cryptic plasmid was termed pNK29-3 and had a low GC content of 33.4%. Two open reading frames were identified on the plasmid, which encode putative mobilization and replication proteins (Figure S2). By comparing the coverage depth of the plasmid to the average coverage depth of the genome, we estimate the copy number to be around nine plasmids per cell. BLAST-analysis revealed a high resemblance to the pIGMS31 previously isolated from *Klebsiella pneumoniae* as well as pEA1 (DQ659147.1) isolated from a Brazilian *Pantoea agglomerans* strain (Figure S2; Smorawinska et al., 2012; Carattoli et al., 2014). It was shown that while pIGMS31 can be mobilized to Alpha- and Gamma-proteobacteria, it replicates via a rolling circle mechanism that functions in Gammaproteobacteria only (Smorawinska et al., 2012). Therefore, pNK29-3 likely originates from other Gammaproteobacteria constituents of the gut flora.

Coinciding with the acquisition of pNK29-3 by lineage A, the 6 m_2_ and 12 m isolates of lineage A were also missing ~54 kb of their chromosome compared to the previous isolates. Annotations and flanking attR and attL sites of this region suggested that it was an integrated phage. Using the PHAST server and BLAST searches against NCBI GenBank, we detected a high similarity to the Siphoviridae prophage of *E. coli* strains FH199 and UMNK88 (Zhou et al., 2011). Losing this prophage did not alter the resistance profile of the strain, but might have been a result of negative selection imposed by increased excision activity as a consequence of the cellular stress imposed by antibiotic exposure (Beaber et al., 2004).

### Lineage a harbored a highly disseminated and stable virulence plasmid

**Figure 3:**
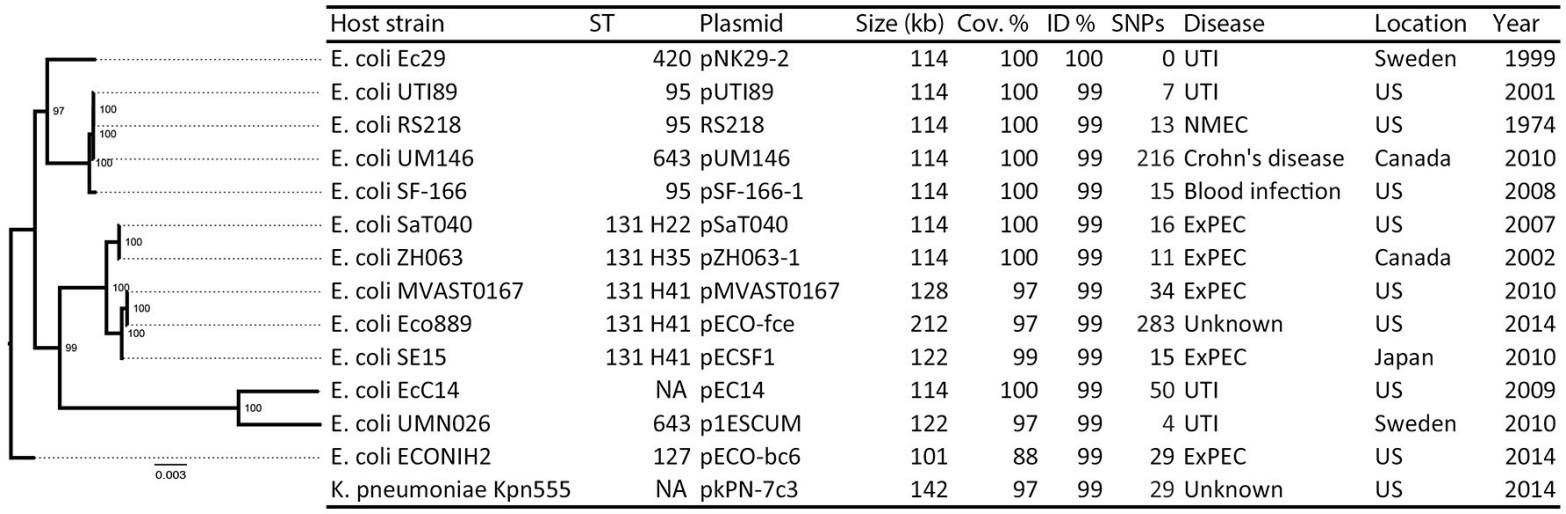
**Overview of plasmids resembling pNK29-2 and their host phylogeny**. Thirteen plasmids with high similarity to pNK29-2 were downloaded from Genbank (see Table S4 for accession numbers and references). Info on host strain, its disease associations and geographical origin was obtained from the literature. The “Year” column denotes the first mentioning of the host strain in the literature unless the isolation year was clearly stated. *In silico* MLST typing was performed and FimH types were added to the ST131 clade highlighting their internal diversity. A core-genome based maximum-likelihood tree was constructed to illustrate the diversity of the *E. coli* plasmid hosts. Node numbers are bootstrap confidence values.

The pNK29-2 plasmids of lineage A displayed very high sequence identity to several widely disseminated plasmids deposited in NCBI's Genbank (Figure 3). Interestingly, 12 highly similar plasmids were previously isolated from various pathogenic *E. coli* strains and one originated from *Klebsiella pneumoniae*. In particular, the endemic pUTI89 plasmid was found to have only 7 SNP differences to the pNK29-2 plasmid, and aligning the contigs from this plasmid showed that there were no additional insertions (Chen et al., 2006). Only two of the SNPs lead to non-synonymous changes. These are located in the *rsvB* gene, a resolvase, and in the *traE* gene, a conjugal transfer protein for F pilus assembly (Table S3). We tested the ability of pNK29-2 to conjugate to *E. coli* MG1655, in liquid and on solid media, and we did not detect any transconjugants; implying that the mutated *traE* transfer gene is dysfunctional in pNK29-2.

### pNK29-2 resembles virulence plasmids found in a diverse set of pathogenic *E. coli*

The *E. coli* strain UTI89, harboring the pUTI89 plasmid, is an archetypical uropathogenic *E. coli* (UPEC) strain isolated from a patient with an acute bladder infection (Mulvey et al., 2001). The pUTI89 plasmid belongs to the IncFIB/IIA incompatibility group and shares several characteristics with the F-plasmid including a full *tra* operon for conjugative transfer (Chen et al., 2006). Additionally, its core backbone includes stability mediating genes such as the *ccdA*-*ccdB* toxin-antitoxin system and the *stbAB* operon ensuring stable inheritance upon cell division (Cusumano et al., 2010). Several of the lesser conserved plasmid regions can be related to virulence and overall adaptation to the human host. These encode the enterotoxicity (*senB*), copper tolerance (s*csC*/s*csD*) and iron acquisition factors. The *cjrABC* operon encodes proteins involved in iron transport that also cause sensitivity to colicin and has shown involvement in UTI virulence (Smajs and Weinstock, 2001; Cusumano et al., 2010).

While a majority of the bacterial hosts carrying these virulence plasmids is associated with UTIs, extremely similar plasmids have been isolated across *E. coli* patho- and sequencetypes from all over the world (Figure 3). For example, the prototype neonatal meningitis *E. coli* strain RS218, isolated in 1974, carries a virulence plasmid virtually identical to pUTI89 and pNK29-2, which has been shown to play an important role in its pathogenicity (Wijetunge et al., 2014).

Likewise, the genomic backgrounds hosting these plasmids are diverse (Figure 3) and include strains of the dominating extraintestinal pathogenic *E. coli* ST131 of major clinical importance (Stoesser et al., 2016). The geographical locations where these strains have been isolated varies, with highly similar plasmids isolated from different strains in the US, Japan, Canada, and Sweden, suggesting that these pUTI89-like plasmids are globally disseminated in a non-clonal fashion (Figure 3).

### Plasmids similar to pNK29-2 carry antibiotic resistance genes obtained from independent insertion events

**Figure 4:**
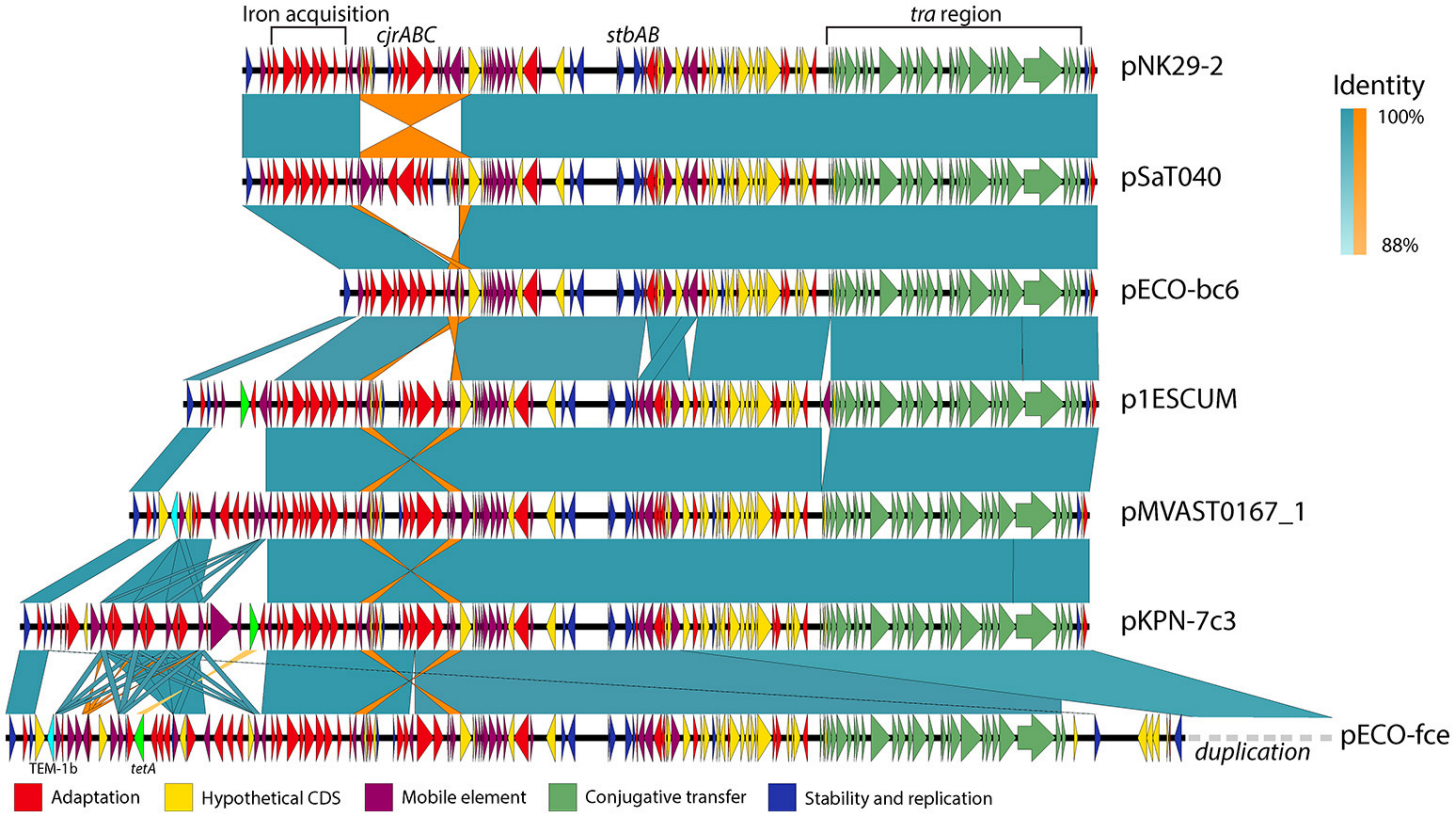
**Genetic variability and conservation within similar virulence plasmid backbones**. six out of the Thirteen virulence plasmids similar to pNK29-2 showed signs of major restructuring events and are illustrated here. While these plasmids show some degree of variation, they also share a conserved core of transfer (green), stability (blue), and virulence genes (red). Mobile elements constituting inverted repeats (highlighted in orange) allow for instability of the *cjrABC-*containing region exemplified in pSaT040 and pECO-bc6. Additional genes involved in antibiotic resistance have been inserted upstream the fully conserved iron acquisition cluster of the genetic load region in p1ESCUM, pMVAST0167_1, pKPN7c3, and pECO-fce. The TEM-1b and *tetA* genes are highlighted in cyan and light green respectively. pECO-fce is significantly larger than the remaining plasmids due to a duplication of its transfer region which has been condensed for simplicity. Colored shades illustrate BLAST identity of pairwise comparisons and orange shades highlight inverted regions.

Although these plasmids share substantial homology, some show variation within defined but variable “genetic load” regions of their plasmid backbone (Figure 4).

Antibiotic use is a strong selection force and some of the plasmids similar to pNK29-2 encode one or more, antibiotic resistance genes organized among mobile element associated cassettes. For instance, the p1ESCUM and pKPN-7c3 plasmids have acquired the *tetA* gene, conferring tetracycline resistance, at different positions within their genetic cargo region, but associated with the same mobile elements (Figure 4; Johnson et al., 2016). pMVAST0167_1 and pECO-fce encode multiple antibiotic resistance genes from their genetic load region and share a TEM-1b gene in the same location and accommodate a similar integron with genes conferring resistance toward aminoglycosides (*aadA5*), sulphonamides (*sul1*) and trimethoprim (*dfrA17*). Compared to pMVAST0167_1, pECO-fce encodes several additional resistance genes (*sul2, tetA, strA, strB*, and *tmrB*) from a cassette inserted between the TEM-1b gene and the *int1* integron (Figure 4).

### pNK29-2 confers a fitness cost to its host *In vitro*

While the pNK29 plasmid, encoding the TEM-1b β-lactamase, was strongly selected for during the β-lactam treatment administered here (Karami et al., 2007), it is not obvious how large virulence plasmids persist in the gut. The lineage A strain persistently colonized the gut for the entire duration of the study, from 2 days to 1 year after birth, suggesting that it is well adapted to the human host. Such long term survival could be supported by general persistence factors of UPEC strains, suggesting an overlap between UTI virulence and gut persistence factors (Nowrouzian et al., 2005; Chen et al., 2013). Only minor selection is required for a plasmid to survive if it does not impose a significant fitness cost on its host. We used antibiotic resistance markers to tag the plasmid and the host chromosome of lineage A in order to separate the plasmid-bearing variant from the cured variant and detect plasmid loss as well as transfer events. We used markers previously used for tagging in similar fitness experiments, where they did not impose a measurable cost (Chen et al., 2013). Similarly, we also confirmed that the Sh *ble* Zeocin resistance gene, used for plasmid tagging, did not impose a significant cost during 5 days of competitive growth against the non-tagged variant (two sample *t-*test, *P* = 0.11).

The cured and plasmid bearing strains were mixed in equal proportions and propagated in LB medium for 8 days without selection. From CFU quantifications on selective agar plates, the average *in vitro* fitness cost of the plasmid-carrying strain was measured to be 0.92 sd ± 0.25% per generation. Although this cost of plasmid carriage is low, one would expect a steady decline of the plasmid bearing cells under these growth conditions. Due to the presence of putative iron acquisition systems on the pNK29-2 plasmid, we also tested the competitive fitness as well as absolute growth rates in iron-limited M9 minimal medium containing 5 μM FeCl_3_ mimicking the lower range of physiological concentrations (Wang, 1996). We were not able to detect a significant advantage in terms of growth rate (Two sample *t-*test, *P* = 0.43—Figure S3) or competitive fitness of the plasmid carrying strain when mixed 1:1 in the same medium (1.07 ± 2%, one sample *t-*test, *P* = 0.45).

### The plasmid carrying strain is outcompeted *In vivo* of the mouse gut

**Figure 5:**
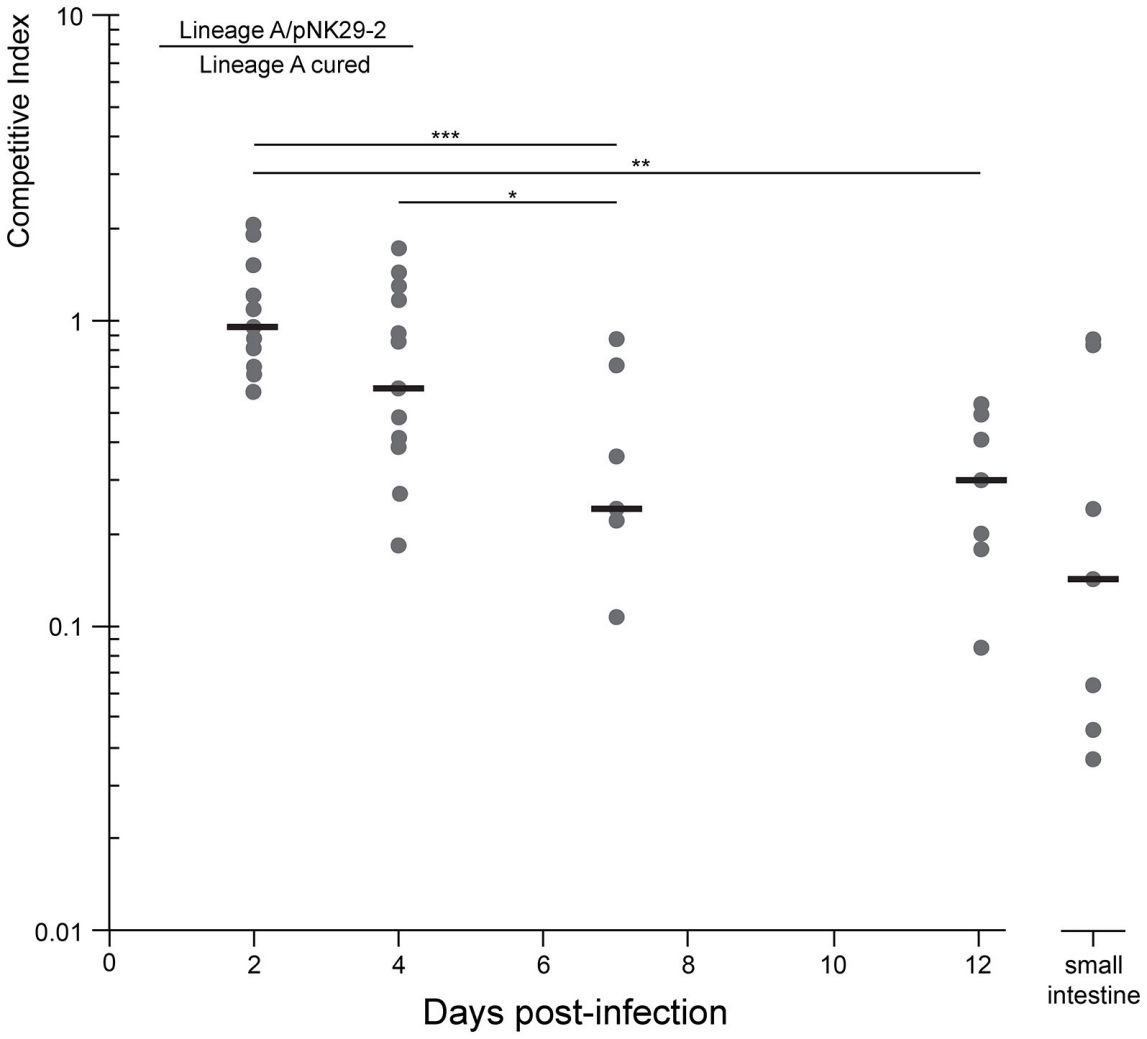
**Competitive growth of lineage a strain against a pNK29-2 cured variant within streptomycin treated mice**. Mice where inoculated by oral gavage with equal amounts of plasmid carrying and plasmid-free cells. The competitive index (top left equation) is the ratio of CFUs obtained from selective plating. This ratio was also quantified for the small intestine at the endpoint (day 12) of the experiment to assess the potential role of adhering cells. Replicates were excluded when the strains were no longer detected. Significance indicators illustrate pairwise comparisons of means (Mann-Whitney *U***-**Test: ^*^P = 0.014, ^**^P = 0.0015, ^***^P = 0.001)

Because the plasmid encodes a diverse set of factors thought to be involved in iron acquisition, toxin production as well as several hypothetical proteins, that might be selected for in more complex *in vivo* settings, we set out to test the competitive fitness in a gut environment of streptomycin treated mice. The plasmid-cured strain was competed against its plasmid-carrying ancestor in the mouse gut and we measured the proportion of plasmid-carrying to plasmid-free cells in the feces over the course of 12 days (Figure 5). Here, we observed a steady decline in plasmid-carrying cells compared to plasmid-free cells with the plasmid-free cells dominating the average population after 7 days of direct competition. Interestingly, the plasmid-carrying populations avoid complete out-competition by plasmid-free cells in this time-span (Figure 5).

To investigate whether the measurements obtained from feces were representative of the intestinal contents, including cells adhering to the intestinal wall, we measured the proportion of plasmid-bearing cells sampled directly from the small intestine of the mice at the end of the 12 days experiment. There was no significant difference between the direct sampling compared to fecal counts (Mann-Whitney *U*-Test, *P* = 0.27) and >10% of the cells contained the plasmid in the average replicate population at this stage (Figure 5).

From the competition experiment we calculated the average fitness cost per generation of the plasmid in the mouse gut to be 0.83 *sd* ± 0.2%, assuming that the competing *E. coli* underwent 10 generations per day (Rang et al., 1999; Lee et al., 2010; Myhrvold et al., 2015). This cost is slightly lower, but not significantly different, from the plasmid cost measured *in vitro* (Two sample *t*-test: *P* = 0.61).

### pNK29-2 is stably inherited despite its cost

Although the plasmid-bearing strain is less fit than the plasmid-cured variant *in vivo*, it seems possible that a minor subpopulation of plasmid-bearing cells can persist for extended time periods; especially if competition from plasmid-free daughter cells is postponed via stable inheritance mechanisms. As for all the virulence plasmids analyzed here (Figure 3), pNK29-2 carries the highly conserved *stbAB* stability operon (Figure 4) that encodes active segregation machinery; ensuring that the plasmid is stably segregated to both daughter cells during cell division (Guynet et al., 2011). To test the segregational stability of pNK29-2 in its native lineage A host (day 0 isolate), we conducted 14 days of serial passaging in LB medium, corresponding to ~140 generations of growth. In such a setup, plasmid-free segregants would eventually take over the population due to the cost of plasmid carriage. However, we did not observe any plasmid-free cells within this time span with a detection limit of plasmid-free cells of ~1%. Similarly, no plasmid-free cells were detected in the *in vivo* competition assays, during the 12 days of gut colonization.

Taken together, these results imply that virulence plasmids, such as pNK29-2, have no direct advantage in gut colonization but are able to persist in spite of a small, but significant, fitness cost due to efficient plasmid inheritance mechanisms.

## Discussion

Culture independent methods based on metagenomic sequencing have been used to investigate the abundance profiles of strains colonizing the gut over time (Morowitz et al., 2011; Brown et al., 2013; Sharon et al., 2013). However, these methods are limited in their ability to observe genomic events at high resolution such as horizontal transfer and single nucleotide variations.

Due to our longitudinal sampling and the high resolution of single isolate genome sequencing, we were able to observe a glimpse of the complex genome dynamics of *E. coli* in its native settings. We confirm a gut-inhabiting strain as the origin of a bladder infection; supporting the general belief that UTIs are caused by gut inhabiting *E. coli* strains that eventually enters the urethra (Chen et al., 2013). Furthermore, we report the sequence of pNK29, a novel 42.2 kb IncX plasmid carrying a TEM-1b β-lactamase, which was transferred between the two co-existing *E. coli* lineages of the gut. Few phenotypic reports exist documenting plasmid mediated HGT of antibiotic resistance genes between bacteria in the human gut and our data supports that transfer of resistance genes take place in the gut, and may be enhanced by antibiotic treatment (Bidet et al., 2005; Karami et al., 2007; Trobos et al., 2009; Goren et al., 2010). The recipient lineage was only sampled at one time-point after the termination of β-lactam antibiotic administration (day 32) and declined to undetectable levels thereafter. This could indicate a negative selection of pNK29-carrying lineage B isolates in the absence of antibiotic selection; however, confirming the role of pNK29 in the counterselection of lineage B in the gut will require further *in vivo* competition experiments. Lineage B showed a large drop in population counts when subjected to the first round of trimethoprim treatment upon hospitalization (Figure 1). The prophylactic administration of trimethoprim coincided with the disappearance of lineage B, and could be another likely explanation for its absence in the consecutive time points.

While we did not observe any genomic alterations of lineage B apart from the acquisition of pNK29, lineage A experienced chromosomal deletions and acquired a cryptic plasmid as well as a high number of SNPs during the sampling period (Figure 2).

Genome plasticity is believed to play a crucial role in the adaptation of pathogens to the selective forces imposed by the immune system or the remaining microbiota within a human host (Brzuszkiewicz et al., 2009). The mutation rate observed for lineage A was high and resemble that of mutator phenotypes that are often enriched among UPEC isolates (Labat et al., 2005). Such increased rates of mutation and recombination events might also be the result of antibiotic treatment of the infant; leading to induction of the bacterial SOS response, which has been shown to increase mutation rates (Beaber et al., 2004; Michel, 2005).

Non-synonymous mutations were indeed detected in genes related to antibiotic tolerance, e.g., those involved in folate metabolism (*folA—*targeted by trimethoprim), fusaric acid resistance (*fusB*), ABC-transport and membrane permeability (porins; Table S1). Equivalently, the genomic deletion of the 54 kb region might have resulted from antibiotic mediated stress known to induce prophage excision and increase horizontal gene transfer in general (Beaber et al., 2004; Nanda et al., 2015).

Apart from small cryptic plasmids providing no obvious selective advantage to their bacterial host, gut-inhabiting *E. coli* isolates often carry plasmids that allow adaptation toward the human host by contributing virulence or antibiotic resistance factors (Johnson and Russo, 2005). In addition to the β-lactamase carrying pNK29 IncX plasmid, we identified a 114 kb plasmid (pNK29-2) in lineage A that was strikingly similar to other previously sequenced virulence plasmids from a diverse set of pathogenic *E. coli* strains (Figure 3). These plasmids have been shown to play a role in the initial stages of UTI infection in a mouse model in a different genetic host background, and could have provided lineage A with the necessary virulence factors leading to the successful UTI infection observed in the studied infant. Although it is generally believed that specific pathotypes of *E. coli* carry different virulence plasmids, plasmid backbones virtually identical to pNK29-2 have recently been found in *K. pneumoniae* as well as several divergent *E. coli* strains; with the earliest isolate dating back to 1974 (Figure 3). The high conservation of these plasmids suggests that they provide a universal adaptive benefit to their ExPEC hosts regardless of infection site (Johnson and Nolan, 2009; Cusumano et al., 2010; Wijetunge et al., 2014).

When comparing the genetic composition of the currently sequenced virulence plasmids with high similarity to pNK29-2, it is clear that certain regions tend to preserve genetic features across host, geography and time (Figures 3, 4). These include mediators of iron acquisition, toxin production and putative copper resistance mediators (*scsC* and *scsD*; DebRoy et al., 2010). Carrying genes implicated in virulence, these plasmids could confer a survival advantage to their bacterial host during infection. Prior studies have examined the role of pUTI89 and pRS218 in urinary tract infections and neonatal meningitis, respectively (Cusumano et al., 2010; Wijetunge et al., 2014). These studies did not observe any phenotypic differences *in vitro* between the plasmid bearing and cured host in terms of growth rate, type 1 pilus expression or biofilm formation. However, they did observe a significant difference in infectivity using rodent infection models (Cusumano et al., 2010; Wijetunge et al., 2014).

A vital defense mechanism of the human body is to restrict iron from pathogens, thus acquisition and transport of iron is an important survival mechanism for ExPEC strains *in vivo* (Andrews et al., 2003). Therefore, iron acquisition could be beneficial for survival in many niches of the human body, including the densely populated gut microbiota, where access to iron is limited (Andrews et al., 2003; Nowrouzian et al., 2003). While Cusumano et al. speculated that the *cjr* operon of pUTI89 was beneficial in a UTI infection scenario due to its putative involvement in iron acquisition, we could not detect an advantage in neither absolute growth rate nor competitive fitness of lineage A carrying the pNK29-2 plasmid when grown in iron-limited conditions (Cusumano et al., 2010). Although the effect was small or non-existent in our experimental setups, pNK29-2 might provide an advantage by other means. For example, the pNK29-2 genes encoding siderophore receptors or transporters might provide an advantage, only if the available iron is on a certain form e.g., bound by its respective siderophore. As other iron acquisition systems are located on the chromosome of lineage A, the pNK29-2 encoded systems could be redundant in this strain, but might be selected in other hosts to encourage plasmid maintenance in a communal context.

Previous studies have shown that even minor differences in host genomes can be highly influential in determining plasmid establishment as well as subsequent adaptation and long term persistence (Humphrey et al., 2012; Porse et al., 2016). Thus, it is intriguing that virulence plasmids imposing a minor, but significant, fitness cost without providing any strongly selected phenotypes, can persist in a competitive environment such as the human gut. From our *in vivo* competition experiment, it is clear that the pNK29-2 carrying strain was less fit in the murine gut but does reach stable counts from day 7 to 12 (Figure 5). This stagnation in competition could encourage extended plasmid persistence and might be explained by changes in the growth rate of gut inhabiting strains over time; indicating a non-constant selection pattern (Rang et al., 1999). Such selection patterns are known to occur in mixed bacterial populations encompassing social iron acquisition phenotypes and similar dynamics could take place in the competition between pNK29-2 carrying and plasmid-free strains in the gut (Stojiljkovic et al., 1993; Ross-gillespie et al., 2007). However, this does not seem to be the case from our growth rate measurements in iron-limiting conditions, were the plasmid carrying strain did not have an advantage on its own (Figure S3).

The measured fitness cost of the pNK29-2 in the lineage A strain was surprisingly similar between *in vitro* and *in vivo* setups, and is low compared to previous observations of plasmid costs (Vogwill and MacLean, 2015). While plasmids can in theory compensate their loss and fitness cost by re-infection of plasmid-free hosts (Slater et al., 2008), this is unlikely to be the case for pNK29-2 as we did not observe any transconjugants in our *in vitro* conjugation assays nor during the *in vivo* competition experiment. The inability of plasmids to conjugate might be explained by the fitness constraints a functional conjugation machinery can impose on certain hosts; supported by the existence of plasmid variants, such as pCE10A from Lu et al., lacking the conjugative transfer operon, that are otherwise identical to pNK29-2 (Lu et al., 2011; Porse et al., 2016).

Loss of pNK29-2 was not observed, neither *in vivo* nor *in vitro*, suggesting that primary stability mechanisms such as active segregation and toxin-antitoxin systems are the most important persistence parameters for these plasmids. This is consistent with the high degree of conservation of stability systems among all 14 plasmids examined here and further supported by the fact that we, as well as previous studies, have experienced considerable trouble curing strains from these plasmids; unless the *stbAB* operon is disrupted (Cusumano et al., 2010; Wijetunge et al., 2014).

By characterizing the genomes of persistent lineages of *E. coli* colonizing the gut of an infant, we observed substantial dynamics, highlighting that strains colonizing the human gut undergo continuous change. While genomic plasticity can lead to improved persistence, some elements are surprisingly stable. Our cost and stability characterizations suggest that a low cost and a high segregational stability, combined with plasmid-encoded universal virulence factors, presumed to increase fitness in a broad range of infection scenarios (Cusumano et al., 2010; Wijetunge et al., 2014), are likely the main parameters governing the success of endemic virulence plasmids. Further understanding of the factors contributing to genomic variation of gut colonizing pathogens will aid in rational interventions against the virulence and antibiotic resistance determinants widely disseminated among these isolates.

## Data availability

All sequenced genomes can be accessed via the Bioproject PRJNA352659.

## Author contributions

NK, IA, and AW provided the *E. coli* isolates. AP did the *in vitro* work and strain tagging. JK performed the *in vivo* competition experiment. HG processed the sequencing data. AP, HG, and MS analyzed the sequencing data results and AP and HG wrote the manuscript with input from MS, JK, DA, NK, and IA.

## Funding

This research was funded by the EU H2020 ERC-20104-STG LimitMDR (638902) and the Danish Council for Independent Research Sapere Aude programme DFF -4004-00213, the Medical Faculty of the University of Göteborg (ALFGBG138401) and the Swedish Medical Research Council.

### Conflict of interest statement

The authors declare that the research was conducted in the absence of any commercial or financial relationships that could be construed as a potential conflict of interest.

## Supplementary material

Figure S1 **Plasmid map and BLAST comparison of pNK29**. pNK29 compared to IncX1 plasmids pOLA52 (outer ring, green) and pRPEC180_47(middle ring, blue). Open reading frames are drawn directionally (inner ring, black). Selected annotations are labeled outside the ring (see Table S2 for full annotation list).

Figure S2 **Plasmid pNK29-3 compared to most similar plasmids in GeneBank**. The two ORFs encode putative replication and mobilization proteins and open reading frames are drawn directionally (inner ring, black).

Figure S3 **Growth rate of lineage A with and without pNK29-2 under iron-limited conditions**.

Table S1 **SNPs in different isolates of lineage A**. Table containing the SNPs from lineage A, including the annotation and whether the animo acid change was synonymous or non-synonymous. The isolates from days 2, 9, and 16 are left out as they did not have any SNP differences from the representative genome for the lineage (isolate taken at 2d).

Table S2 **Annotations for pNK29**. Names in square brackets indicate homologs in pOLA52.

Table S3 **SNPs in the pNK29-2 plasmid of lineage A compared to pUTI89**. SNPs were identified by comparing the initial linage A isolate (day 2) with pUTI89 (CP000244.1).

Table S4 **Plasmids similar to pNK29-2 and information on their origin**. The table presented in Figure 3, but more detailed information on size, location, and references. The GenBank ID refers to the plasmid sequence entry in GenBank and the Pubmed ID (PMID) to the study where the plasmid was initially described.
