# Supplementary figures and images for: Genome Dynamics of Escherichia coli during Antibiotic Treatment: Transfer, Loss, and Persistence of Genetic Elements In situ of the Infant Gut
Source: Front Cell Infect Microbiol. 2017 Apr 12;7:126. doi: 10.3389/fcimb.2017.00126 (PMC5388698; doi:10.3389/fcimb.2017.00126)

pNK29

PEC180\_47

100% identity

90% identity

80% identity

A52

100% identity

90% identity

80% identity

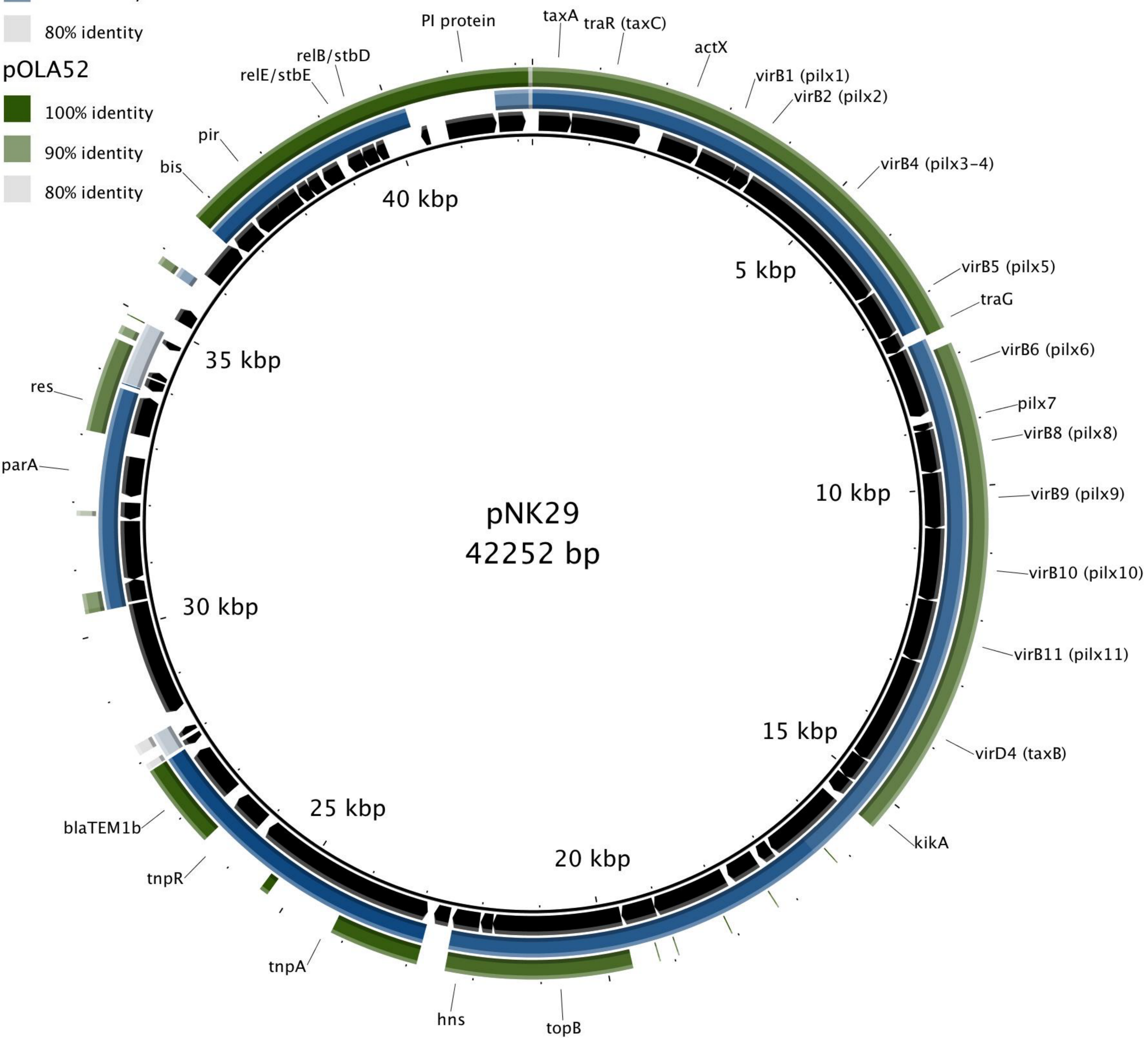

Supplement: Figure S1 — Plasmid map and BLAST comparison of pNK29. pNK29 compared to IncX1 plasmids pOLA52 (outer ring, green) and pRPEC180_47(middle ring, blue). Open reading frames are drawn directionally (inner ring, black). Selected annotations are labeled outside the ring (see Table S2 for full annotation list). [file Image1.PDF]

■ pNK29-3

pEA1

■ 100% identity

■ 90% identity

■ 80% identity

pIGMS31

■ 100% identity

■ 90% identity

■ 80% identity

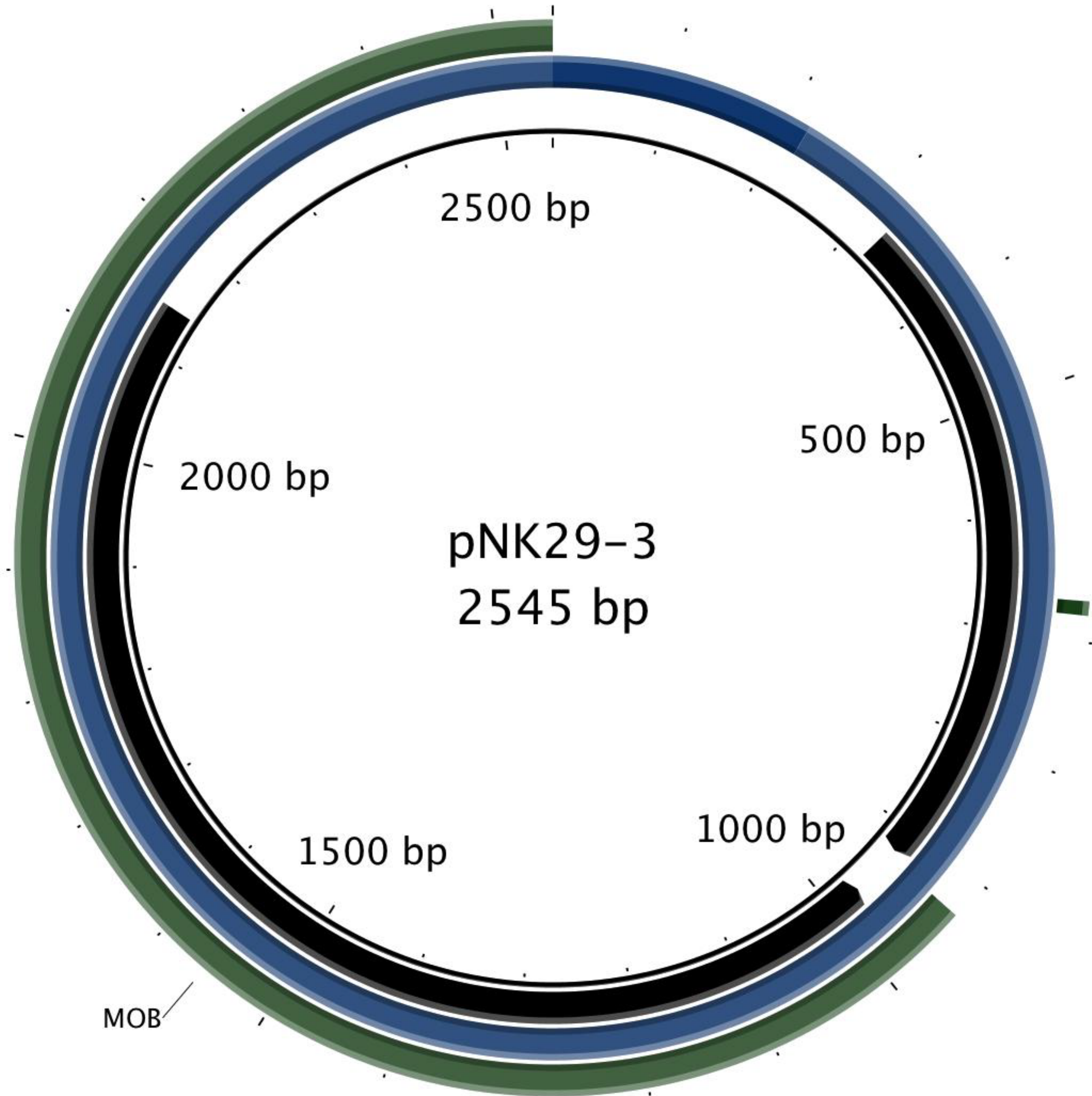

Supplement: Figure S2 — Plasmid pNK29-3 compared to most similar plasmids in GeneBank. The two ORFs encode putative replication and mobilization proteins and open reading frames are drawn directionally (inner ring, black). [file Image2.PDF]

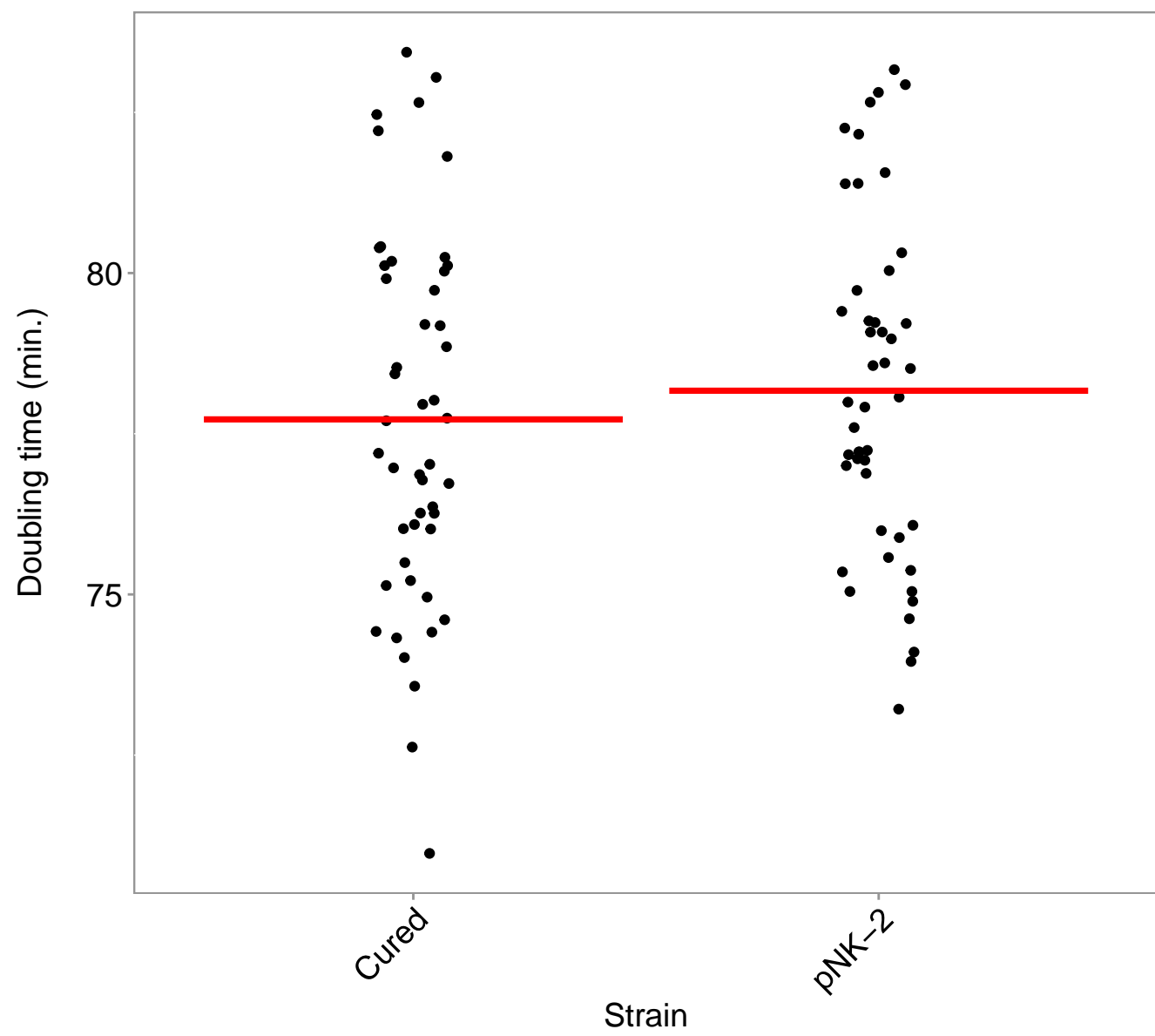

Supplement: Figure S3 — Growth rate of lineage A with and without pNK29-2 under iron-limited conditions. [file Image3.PDF]
